# Diagnostic Applications of Artificial Intelligence in Liver Diseases

**DOI:** 10.3390/jcm14176231

**Published:** 2025-09-03

**Authors:** Maria Consiglia Bragazzi, Rosanna Venere, Gloria Andriollo, Lorenzo Ridola, Domenico Alvaro

**Affiliations:** 1 Department of Medical and Surgical Sciences and Biotechnologies, Sapienza University of Rome, 04100 Latina, Italy; rosanna.venere@gmail.com (R.V.); gloria.andriollo@uniroma1.it (G.A.); lorenzo.ridola@uniroma1.it (L.R.); 2 Department of Translational and Precision, Sapienza University of Rome, 00161 Rome, Italy; domenico.alvaro@uniroma1.it

**Keywords:** artificial intelligence, deep learning, chronic liver disease, cirrhosis, focal liver injury

## Abstract

Artificial intelligence encompasses the capacity of machines to emulate human faculties, including reasoning, learning, planning, and creativity. Presently, chronic liver disease, liver cirrhosis, primary liver tumors, and liver metastasis represent significant and escalating causes of mortality worldwide. Consequently, there is a growing demand for more efficient and minimally invasive tools to enhance the diagnostic and therapeutic approaches for these ailments. Over recent years, endeavors have been directed towards employing artificial intelligence within hepatology to advance the diagnosis and treatment of liver diseases. This paper aims to outline the latest developments in the application of artificial intelligence within the realm of liver disease.

## 1. Introduction

Artificial intelligence (AI) involves all operations and functions that characterize the intellectual capabilities of humans and can be executed with the assistance of computers. These include planning, language comprehension, object and sound recognition, learning, and problem solving. Machine learning (ML) is a specific application of AI that focuses on machines’ ability to receive data and learn autonomously, progressively modifying algorithms based on the information they process. Commonly used algorithms include logistic regression, decision trees, random forests, and deep neural networks, depending on the clinical application. These models are trained using techniques such as cross-validation, regularization, and, when necessary, transfer learning, to ensure accurate and generalizable predictions.

**Figure 1:**
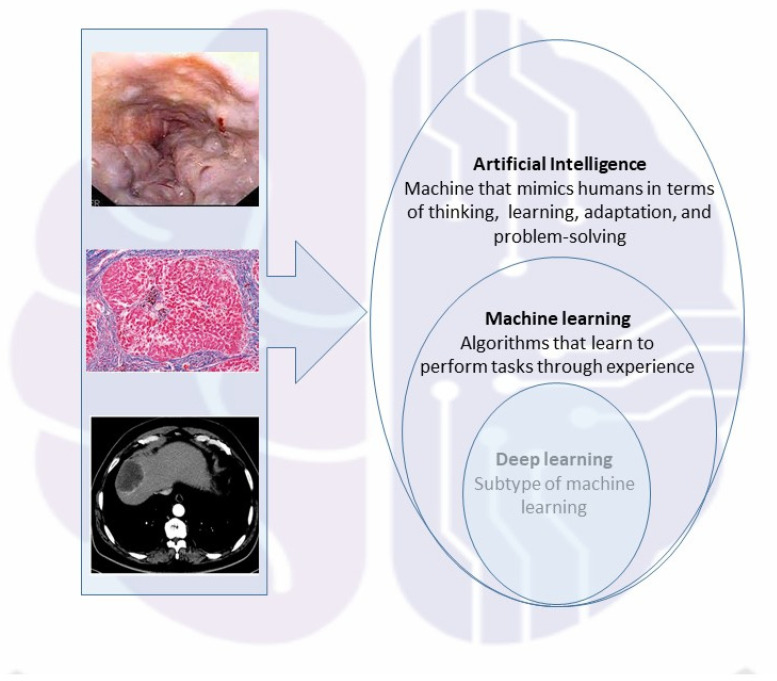
Schematic representation of AI comprising both ML and DL enclosed in concentric sets, with applications spanning various fields of human health.

The terms AI and ML are often used interchangeably, especially in the realm of big data. ML utilizes methods such as neural networks, statistical models, and operational research to uncover hidden information in data. Deep learning (DL) is an approach to ML inspired by the structure of the brain, characterized by the interconnection of various neurons. DL employs models of neural networks with multiple processing units, leveraging computational advances and various training techniques to learn complex patterns through the analysis and processing of vast amounts of data. Thus, AI encompasses both ML and DL in a representation of concentric sets (Figure 1), with applications that span numerous fields in human health.

The extensive application of AI not only contributes to improving the diagnosis and treatment of liver diseases but also plays a central role in liver research and its pathologies. Artificial neural networks have been created in recent years, significant progress has been made in regulatory and legislative fields, leading to the possibility that AI will overcome current biases and soon be fully integrated into everyday clinical practice.

Translational hepatology is a research area that primarily focuses on studying liver diseases and their treatment, with particular attention to translating scientific findings from basic research into practical applications to address unmet needs in a broad spectrum of liver diseases, from viral hepatitis to liver cirrhosis and primary liver tumors. However, numerous questions remain open. AI is believed to provide effective insights into future epidemiological developments in liver diseases, develop economically advantageous and cost-effective solutions for diagnosing liver diseases, and help understand the causes and risk factors predisposing the progression of liver fibrosis, cirrhosis, hepatocellular carcinoma (HCC), or cholangiocarcinoma (CCA). Additionally, AI can assist clinicians or surgeons in choosing the most effective treatment for various liver diseases, identify the most efficient treatments to slow the progression of liver cirrhosis towards decompensation and HCC, and finally, develop predictive models for effective liver allocation in transplantation planning [1,2,3,4].

## 2. Materials and Methods

This review was based on a literature search using PubMed (English language), Google Scholar, and Web of Science. We included relevant studies that investigated the use of artificial intelligence (AI), machine learning (ML), deep learning (DL), or radiomics (that is, the extraction of quantitative features from medical images to help improve diagnosis and treatment decision) in the diagnosis, staging, and management of liver diseases. We selected original articles, systematic reviews, and meta-analyses focusing on non-invasive imaging (e.g., ultrasound, CT, MRI, elastography), histopathology, or clinical data analysis. The relevant data were narratively presented. Findings were summarized in comparative tables to facilitate interpretation and highlight the evolving role of AI in hepatology.

## 3. Artificial Intelligence and Chronic Liver Diseases

Chronic liver diseases and hepatic cirrhosis are currently significant causes of death worldwide. Evaluating the stage of fibrosis is crucial for establishing the prognosis, treatment, and follow-up of patients with liver disease. Liver biopsy is the gold standard for defining the stage of fibrosis; however, over the years, numerous non-invasive methods have been identified to stage fibrosis, including the use of serum markers (FIB-4 and APRI), techniques reflecting the physical properties of the liver such as liver stiffness (transient elastography and shear-wave elastography), and methods based on imaging (CT, MRI, ultrasound (US)) [5].

Imaging techniques such as CT, MRI, and US are fundamental for evaluating complications related to chronic liver diseases, and the use of AI allows extracting information from a vast amount of multi-parametric clinical data. From the literature, for example, we know that the nodular/irregular appearance (LSN score) of the liver tends to increase with the progression of cirrhosis; such appearance is easily detectable with the main imaging techniques. Based on these premises, various semi-automatic tools have been developed, demonstrating high accuracy in identifying cirrhosis among various CT sequences (area under the curve (AUC) 0.902 for significant fibrosis, 0.932 for advanced fibrosis, and 0.959 for cirrhosis) [6].

Recently, AI has also been applied to MRI for staging liver fibrosis. In 2017, in a retrospective study by Park et al., they evaluated the diagnostic performance of a deep convolutional neural network (DCNN) model, in staging liver fibrosis in the hepatobiliary phase with gadoxetic acid, in patients with HBV and HCV-related liver diseases. They compared the data obtained with the histological results from biopsies and/or surgical samples. The fibrosis score obtained (FDL score) was significantly correlated with the stage of fibrosis; they were diagnosed with an area under the ROC curve (AUROC) of 0.84 (F4), 0.84 (F3), and 0.85 (F2), respectively [7]. In 2019, Lili He et al. developed an ML model that detected liver fibrosis derived from MR elastography (MRE), aligning with clinical MRI characteristics and MRI without elastography by categorizing clinical and radiomic features separately or using both. The use of a support vector machine (SVM) showed an accuracy of 75.0%, a sensitivity of 63.6%, and a specificity of 82.4%. This demonstrated that this model, with the assistance of radiomic features weighted in T2, can contribute to the diagnosis of even minimal liver fibrosis [8]. Their results were internally and externally validated by training and testing their SVM model on MRI scanners from two different manufacturers. In 2020, Schwhawk demonstrated, instead, the accuracy in quantifying elevated liver fibrosis similar to MR elastography, using parameters based on texture analysis (TA) employing an ML approach applied to T1- and T2-weighted images [9]. This prospective study suggests that liver fibrosis can be assessed with TA-derived parameters of T1-weight when combined with an ML algorithm with similar accuracy compared to MRE.

In US imaging, the use of AI has also proven to be significantly accurate and an alternative to traditional elastography in predicting the stage of fibrosis. This was made possible through a DCNN model that achieved an AUC for the diagnosis of cirrhosis of 0.90 on the internal test set and 0.857 on the external test set, respectively, confirming better performance even compared to data produced with traditional US by five radiologists [10].

Wang K et al., on the other hand, evaluated the performance of a DL model (DLRE) for assessing stages of liver fibrosis, which involves using radiomics for quantitative analysis of heterogeneity in two-dimensional shear wave elastography (2D-SWE) images, in relation to APRI and FIB-4. The AUCs of DLRE were found to be 0.97 for F4, 0.98 for ≥F3, and 0.85 for ≥F2, demonstrating that this model performs better in predicting stages of liver fibrosis compared to 2D-SWE and biomarkers [11]. Similar results were also demonstrated by the group of Gatos I et al., who, through the use of an ML algorithm applied to SWE, reduced the variability between measurements, thereby improving the classification of various fibrosis stages [12] (Table 1).

However, the prospective multicenter study by Wang K et al. was limited to HBV patients and required multiple SWE acquisitions, which raises concerns about its applicability in routine practice. Likewise, the work of Gatos I et al., though promising, relied on hand-crafted features and lacked external validation, leaving uncertainty about its robustness. These examples show that even very accurate AI models in research may still face challenges in proving their value in real-world hepatology.

The use of AI in the non-invasive diagnosis of portal hypertension and gastroesophageal varices has proven to be highly valuable in clinical practice. Importantly, this model underwent both internal and external validation, which reduces the risk of overfitting and enhances the generalizability of the findings. As highlighted in the recent review by Yu Q. et al., the use of meta-algorithms created from demographic, clinical, laboratory, instrumental, and transient elastography measurements has enabled the detection of HVPG ≥10 mmHg with a precision of 89.72%, an AUC of 0.96, a PPV of 86.13%, and an NPV of 45.45%. While the high AUC indicates a strong discriminative performance compared to the gold standard, the PPV and NPV provide additional clinically relevant insights, supporting the model’s utility in guiding decisions when invasive HVPG is not available. Importantly, this model underwent both internal and external validation, which reduces the risk of overfitting and enhances the generalizability of the findings. Thanks to ML, it has also been possible to identify an “EVendo score” formula, which could avoid unnecessary gastroscopies in patients at low risk of esophageal varices but at high risk of bleeding [15]. Another real-time DCNN system has been discovered and validated by Wang J et al., called ENDOANGEL. This system was designed to diagnose gastroesophageal varices and predict the risk of rupture. Compared to the assessment by endoscopists, it was found to have an accuracy of 97.00% and 92.00% in terms of detecting esophageal varices and gastric varices, respectively [16]

Contrary to liver cirrhosis and viral hepatitis, whose global prevalence is progressively decreasing, nonalcoholic fatty liver disease (NAFLD), nonalcoholic steatohepatitis (NASH), and related cirrhosis are progressively increasing, with an estimated prevalence of around 33.5% by 2030 [17].

The gold standard for diagnosing NAFLD/NASH is represented by liver biopsy. In recent years, the application of AI in this patient setting has become increasingly widespread for the purpose of diagnosing and quantifying fibrosis and inflammation. Multidisciplinary clinical models and the creation of electronic health records (EHR) containing patient information, such as sex, body mass index, laboratory tests, etc., have enabled the development of AI and ML systems. These systems are capable of diagnosing NASH, for example, in patients with NAFLD, as demonstrated by Fialoke et al. in 2018, who used the ML method with an AUROC of 88% [18]. However, while this approach highlights the potential of ML using routine clinical data, its retrospective design and lack of external validation may limit generalizability.

The use of AI has proven effective even in comparison to histology in quantifying fibrosis, ballooning, steatosis, and inflammation in patients with NASH. Liu et al. with an AI system called qFibrosis, qBallooning, qInflammation, and qSteatosis (qFIBS) showed a strong correlation with each element of the NASH Clinical Research Network qFBIS score with high AUROC values, demonstrating the ability to distinguish different stages of the disease [19]. qFIBS showed excellent concordance with pathologists and holds promise for advancing NASH assessment despite its current reliance on biopsy. The group of Forlano R et al. also used a fully automated ML system for quantifying inflammation, steatosis, ballooning, and fibrosis in biopsy samples from patients with NAFLD, achieving a concordance between ML and the observer (pathologist) ranging from 0.95 to 0.99, underscoring its potential to enhance the accuracy and consistency of histological assessment, though still limited by single-center validation [20]. Another demonstration of accuracy in measuring the heterogeneity, severity, and treatment response of NASH was introduced by Taylor-Weiner et al. using an ML method called PathAI to quantify liver histology and monitor disease progression in NASH based on biopsy samples from three randomized controlled trials [21]. This system has proven to be a valuable aid for researchers in classifying NASH patients at high risk.

## 4. Artificial Intelligence and Focal Liver Lesions

ML has also found utility in the diagnosis of primary liver tumors. It has been observed that ML has achieved excellent performance in diagnosing and radiologically classifying potentially malignant liver lesion [22,23,24,25].

It has been noted that DL based on convolutional neural networks (CNNs) can achieve such capabilities by analyzing images with and without disease. In a preliminary study, Hamm et al. developed and evaluated the ability of CNN-based DL to distinguish six common liver lesion types (simple cysts, cavernous angiomas, focal nodular hyperplasia (FNH), HCC, and liver metastases secondary to colorectal cancer) with typical imaging features on multiphasic MRI. A total of 494 liver lesions with imaging features typical of the six liver lesion classes mentioned above were examined. A subset of each lesion class was labeled with up to four key imaging features per lesion. Feature maps highlighting regions of the original image corresponding to particular features were generated, and relevance scores were assigned to each identified feature. The interpretable DL system achieved a sensitivity of 82.9% and a positive predictive value (PPV) of 76.5% in identifying radiological features annotated by expert readers across different lesion types. While performance varied by feature complexity, key diagnostic features such as arterial phase hyperenhancement were identified with PPVs exceeding 90%. These results demonstrate the system’s potential to provide interpretable insights into CNN decision making by linking lesion classification to specific, quantifiable imaging features [23].

Studies have also been conducted to apply AI as decision support in classifying liver metastases according to their primary tumor origin. Currently, once a liver metastasis is detected, identifying the primary tumor is challenging, requiring time and multiple examinations. This is because there are no methods to identify the origin of the metastasis based solely on their appearance in CT image scans. With this premise, Ben-Cohen et al. attempted to create an algorithm to classify liver metastases based on their primary sites using CT examinations as input. The methods were developed using a set of 50 lesion cases extrapolated from 29 patients, while the experiments were conducted on a separate set of 142 lesion cases obtained from 71 patients with the following four different types of primary tumors: melanoma, breast cancer, colorectal cancer, and pancreatic cancer. Each metastasis was evaluated by two radiologists using CT images in the portal phase and in the phase without contrast [26]. Images were analyzed by positioning a region of interest (ROI), which included the boundary of the lesion extracted from each scan input by the radiologist. The computer system proposed in the study recognized several visual features from the input images, with multiple descriptors for each image point, referred to as feature vectors. The generated feature vectors were then input into a classifier, and a leave-one-out cross-validation (LOOCV) technique was performed using “top-n” accuracy, where the N predictions with the highest probability from the model were considered for classification evaluation. While the classification of the four primary tumor sites showed relatively low top-1 accuracy, the results nonetheless exceeded expert performance and demonstrated the potential of imaging-based features to support clinical decision making in this challenging task. However, the system performed better than experts in distinguishing between different metastases. This suggests the potential for new tools in the future that can provide decision making and therapeutic support to radiologists and oncologists, leading to more efficient detection and cancer treatment [26].

Recently, the utility of radiomics in predicting post-therapy response at one year for patients with colorectal liver metastases (CRLMs) was also assessed. Wei S et al. conducted a retrospective study in which they examined patients with CRLMs after one year of bevacizumab therapy. They evaluated the ability of a radiomic model designed to identify the histopathologic growth pattern (HGP) of CRLMs to predict early response and progression-free survival (PFS) in these patients. This was accomplished using multilayer computed tomography with contrast medium (ceMDCT). For each target lesion, radiomically diagnosed HGP (RAD_HGP) was determined, categorizing lesions as desmoplastic (D) or replacement (R) type. Patients with a D pattern had a significantly longer PFS than patients with an R or mixed pattern (*p* < 0.001). RAD_HGP was the only independent predictor of PFS at 1 year. These findings suggest that HGP diagnosed by radiomic pattern could serve as an effective predictor of PFS for patients with CRLM treated with bevacizumab [27].

A similar study was conducted by Dercle et al. in patients with colorectal liver metastases (CRLMs). Radiomic signatures were used during treatment to predict tumor sensitivity to irinotecan, 5-fluorouracil, and leucovorin (FOLFIRI) alone (F) or in combination with cetuximab (FC). A total of 667 patients with these characteristics were retrospectively enrolled. Four tumor imaging biomarkers measured quantitative radiomic changes between standard-of-care CT scans at baseline and at 8 weeks. Using ML, the signature performance was trained and validated using receiver operating characteristic (ROC) curves to classify tumors as sensitive or insensitive to treatment. Cox hazard ratio and regression models assessed the association with overall survival (OS). In sets containing cetuximab, the radiomic signature outperformed existing biomarkers for detecting treatment sensitivity and was strongly associated with OS (*p* < 0.005). This indicates that radiomics-derived supportive tools could provide physicians with early prediction of FOLFIRI treatment success using standard conventional CT scans [28].

Several studies have been conducted on the utility of radiomics in this type of neoplasm. Among these studies, Cheng et al. conducted a retrospective study of 110 patients with metastatic liver lesions secondary to pancreatic neoplasm to assess the effectiveness of radiomics in predicting response. Specifically, they evaluated changes in CT texture of metastatic liver lesions after chemotherapy treatment to determine whether texture parameters correlate with time to progression (TTP). From this study, it was found that the applied criteria were not sufficient for assessing tumor response to treatment. However, the percentage change derived from contrast-enhanced CT texture analysis could better predict the tumor response and TTP in pancreatic cancer patients with liver metastasis [29]. All these studies are limited by their retrospective design, yet their large cohorts, use of standard CT, and advanced analytics strongly support clinical translation.

Shan et al., in their paper, developed a predictive model based on peritumoral radiomic signatures extracted from CT images. The study aimed to assess the effectiveness of radiomic signatures in predicting early recurrence (ER) of HCC after curative treatment. A total of 156 patients with HCC were randomly divided into a training cohort (109 patients) and a validation cohort (47 patients). ER was defined as the presence of new intrahepatic lesions or metastases with typical imaging features for HCC, or atypical features with histologic diagnosis of HCC confirmed within two years after curative surgical resection or ablation. A region of interest (ROI) was manually delineated around the lesion for extraction of tumor radiomic features (T-RO), and another ROI was delineated with an additional 2 cm peritumoral area for extraction of peritumoral radiomic features (PT-RO). T-RO and PT-RO models were constructed using the logistic regression model LASSO (least absolute shrinkage and selection operator) and compared with peritumoral enhancement (PE-T) detected by a radiologist. Comparing AUC values, prediction accuracy in the validation cohort was good for the PT-RO model (0.80 vs. 0.79, *p* = 0.47) but poor for the T-RO model (0.82 vs. 0.62, *p* < 0.01). In the validation cohort, receiver operating characteristic (ROC) curves, calibration curves, and decision curves indicated that the PT-RO model had better calibration efficiency and provided greater clinical benefits. The net reclassification index without categories (CfNRI) indicated that the PT-RO model correctly reclassified 47% of ER patients and 32% of non-ER patients compared with the T-RO model (*p* < 0.01); furthermore, the PT-RO model correctly reclassified 24% of ER patients and 41% of non-ER patients compared with PT-E (*p* = 0.02). This led to the conclusion that the CT-based PT-RO model can effectively predict the ER of HCC and is more efficient than the T-RO model and conventional PE-T imaging function [30]. This work clearly shows the prognostic value of peritumoral radiomics on standard CT, and despite its retrospective, single-center approach and lack of external validation, it provides a strong foundation for future clinical application.

In recent years, an increase in the use of AI to detect and diagnose CCA, the second most frequent primary liver tumor, has been observed. However, the use of AI in evaluating patients with this tumor is not as well-established as that of routine imaging. Radiomic models based on magnetic resonance imaging have performed well in predicting the degree of differentiation (DD) and recognizing lymph node metastasis (LNM) of extrahepatic CCA (ECC), showing significant potential in noninvasive clinical diagnosis and prediction of ECC [31]. Significant results have been obtained from studies conducted through the development of a new radiomic nomogram aiming for early detection of intrahepatic CCA (ICC) recurrence, or to evaluate the ability of the radiomic nomogram to predict the presence of LNM in ICC and biliary tract cancer (BTC), and to determine its prognostic value [32,33] (Table 2).

In the context of ICC, Xu et al. conducted a study to develop a predictive model for preoperative assessment of lymph node (LN) status. They utilized a group of 106 ICC patients for training the prediction model and an independent group of 42 patients for validation. The prediction model was built using a SVM based on features most correlated with LN status, selected using the maximum relevance and minimum redundancy (mRMR) algorithm. An SVM score was calculated for each patient to indicate the probability of LNM, and a combined nomogram incorporating the SVM score and clinical characteristics was constructed. The SVM model utilized five selected image features. The combined nomogram, integrating SVM score, CA 19-9 level, and LNM factor reported by MRI, demonstrated superior discrimination between LNM and non-LNM patients compared to the SVM model alone (AUC: training group: 0.842 vs. 0.788; validation group: 0.870 vs. 0.787). This facilitated personalized assessment of LN status, aiding physicians in making surgical decisions [34].

Radiomics has also been utilized to generate radiomic signatures using US for assessing the biological behaviors of ICC, as well as to evaluate the diagnostic performance of radiomic MRI models in detecting DD and LNM in ECC [32] (Table 2).

Recently, Chu et al. conducted a study on radiomics applied to CT images to preoperatively predict futile ICC resection. In this study, 203 patients with ICC were enrolled from two centers and randomly assigned to the training and validation cohorts at a ratio of 7:3, respectively. The radiomics model demonstrated a higher AUC compared to the clinical model in the validation cohort (AUC: 0.804, 95% CI: 0.697–0.912 vs. 0.590, 95% CI: 0.415–0.765, *p* = 0.043) for predicting futile resection in ICC. The radiomics model achieved a sensitivity of 0.846 (95% CI: 0.546–0.981) and specificity of 0.771 (95% CI: 0.627–0.880) in the validation cohort. These findings led to the conclusion that, when compared to clinical information alone, radiomics utilizing CT images holds greater potential in accurately predicting futile resection before surgery [36].

Therefore, based on recent studies, it can be concluded that the use of artificial intelligence, utilizing US, CT, positron emission tomography (PET-CT), and MRI, for detecting and diagnosing focal liver lesions demonstrates high accuracy (Table 3).

However, there is thus a clear need for large-scale, prospective, multicenter trials to validate these approaches and ensure their safe and effective translation into clinical practice.

## 5. Conclusions and Future Directions

Based on the numerous data available in the literature, AI will be an integral part of clinical practice in the future. The creation of multiparametric systems assisted by AI will indeed improve the diagnostic and therapeutic pathway for patients with chronic liver diseases, liver cirrhosis, and/or primary liver tumors or liver metastases, with the possibility of risk stratification and entirely personalized therapy. Although this review primarily focuses on the application of artificial intelligence in radiological imaging of liver diseases, it is important to acknowledge the growing impact of AI in histopathology. Recent advances in deep learning have shown promising results in classifying and assessing both neoplastic and non-neoplastic liver diseases, sometimes outperforming expert pathologists in specific tasks such as fibrosis quantification and hepatocellular carcinoma subtype identification. Integrating histopathological AI tools with imaging-based approaches may provide a more comprehensive diagnostic framework and represents a valuable direction for future research. Looking ahead, the next steps should prioritize multimodal models that combine imaging with clinical, genomic, pathological, and histological data, as these approaches have already shown superior diagnostic accuracy compared to unimodal frameworks. At the same time, the development of explainable AI systems will be essential to ensure transparency, interpretability, and clinical trust. Equally important will be the establishment of multicenter collaborative networks and harmonized datasets, potentially enriched with synthetic data, to overcome the limitations of sample size and heterogeneity. To confirm the real clinical value of these tools and guide their safe integration into routine practice, carefully designed prospective multicenter studies are required.

## Figures and Tables

**Table 1:** Application of AI in different imaging techniques used for diagnosis and staging of liver fibrosis (with and without mdc).

	AUC ^1^ Significant Fibrosis	AUC Advanced Fibrosis	AUC Cirrhosis	References
Machine learning based on contrast enhanced CT ^2^	0.90	0.93	0.96	Pickhardt P J, 2016 [6]
Deep learning based on contrast enhanced CT	0.96	0.97	0.95	Choi K J, 2018 [13]
Deep learning based on contrast enhanced MRI ^3^	0.85	0.84	0.84	Yasaka K, 2017 [14]
Radiomics based on contrast enhanced MRI	0.91	0.88	0.87	Park H J, 2019 [7]
Deep learning based on US ^4^	0.90		0.90	Lee J H, 2020 [10]
Deep learning based on shear wave elastography	0.85	0.98	0.97	Wang K, 2019 [11]
Deep learning based on shear wave elastography	0.85–0.89	0.845–0.87	0.85–0.87	Gatos I, 2019 [12]

AUC ^1^: area under the curve, CT ^2^: computed tomography, MRI ^3^: magnetic resonance imaging, US ^4^: ultrasound.

**Table 2:** Different application of IA in CCA. The AUC values shown in the table represent the best results from the studies cited above.

Target	N° Patients	Imaging	Metod	AUC ^11^	References
To develop a new radiomic nomogram to predict the ER ^1^ of ICC ^2^.	209	CECT ^3^	LASSO ^4^	0.9	Liang W, 2018 [32]
To develop a radiomic model for predicting the LNM ^5^ of ICC and determine its prognostic value.	103	CECT	LASSO	0.9244	Ji JW, 2019 [33]
To evaluate a radiomic model for predicting LNM in BTC ^6^ and determine its prognostic value.	247	CECT	LASSO	0.81	Ji JW, 2019 [33]
To develop a prediction model for preoperative LNM in patients with ICC	148	MRI	SVM ^7^	0.870	Xu L, 2019 [34]
To develop radiomic signatures based on ultrasound (US) to assess the biological behaviors of ICC 0.930	128	US	LASSO, SVM	0.930	Peng YT, 2020 [35]
To assess the diagnostic performance of radiomic MRI ^8^ models in detecting DD ^9^ and LNM of ECC ^10^.	100	MRI	Random forest	0.9	Yang C, 2020 [31]

ER ^1^: early recurrence, ICC ^2^: intrahepatic CCA, CECT ^3^: contrast-enhanced computed tomography, LASSO ^4^: least absolute shrinkage and selection operator, LNM ^5^: lymph node metastasis, BTC ^6^: biliary tract cancer, SVM ^7^: support vector machine, MRI ^8^: magnetic resonance imaging, DD ^9^: degree of differentiation, ECC ^10^: extrahepatic CCA, AUC ^11^: area under curve

**Table 3:** Artificial intelligence and liver cancer.

Target	Method	Accuracy	References
Predicting the primary origin of liver metastases	CT ^1^-based radiomics and deep learning	56%	Ben Cohen A, 2017 [28]
Detection of new liver tumors	CT-based deep learning	86%	Vivanti R, 2017 [37]
Detection of focal liver lesions	Deep learning based on ultrasound	89%	Tryarattanachai T, 2021 [38]
Detection of liver tumors	Deep learning based on MRI ^2^	90%	Kim J, 2020 [39]
Detection and distinction of different focal liver lesions	Deep learning based on ultrasound	97.2%	Hassan TM, 2017 [40]
Detection of liver metastases	Deep learning based on PET/CT ^3^	90.5%	Yang C, 2020 [31]
Evaluation of focal liver lesions	Deep learning based on MRI	92%	Hamm C, 2019 [23]
Evaluation of focal liver lesions	Deep learning based on CT	84%	Yasaka K, 2018 [41]

CT ^1^: computed tomography, MRI ^2^: magnetic resonance imaging; PET/CT ^3^ positron emission tomography computed tomography.

## Abbreviations

The following abbreviations are used in this manuscript:

AI	Artificial Intelligence
AUC	Area Under the Curve
AUROC	Area Under the Receiver Operating Characteristic Curve
APRI	Aspartate Aminotransferase to Platelet Ratio Index
BTC	Biliary Tract Cancer
ceMDCT	Contrast-Enhanced Multidetector Computed Tomography
CFNRI	Category-Free Net Reclassification Index
CNN	Convolutional Neural Network
CRLM	Colorectal Liver Metastases
CT	Computed Tomography
DCNN	Deep Convolutional Neural Network
DL	Deep Learning
DLRE	Deep Learning Radiomics Elastography
EHR	Electronic Health Record
ENDOANGEL	Endoscopy-Based Artificial Intelligence System for Varices Detection
EV	Esophageal Varices
EVendo	Esophageal Varices Endoscopy Avoidance Score
FIB-4	Fibrosis-4 Index
FNH	Focal Nodular Hyperplasia
HGP	Histopathologic Growth Pattern
HVPG	Hepatic Venous Pressure Gradient
LASSO	Least Absolute Shrinkage and Selection Operator
LOOCV	Leave-One-Out Cross Validation
LSN	Liver Surface Nodularity
ML	Machine Learning
MRI	Magnetic Resonance Imaging
MRE	Magnetic Resonance Elastography
mRMR	Maximum Relevance Minimum Redundancy
PathAI	Pathology-Based Artificial Intelligence System
PE-T	Peritumoral Enhancement
PET-CT	Positron Emission Tomography Computed Tomography
RAD_HGP	Radiomically Diagnosed Histopathologic Growth Pattern
ROC	Receiver Operating Characteristic
ROI	Region of Interest
SVM	Support Vector Machine
T-RO	Tumoral Radiomic Features
TTP	Time to Progression

## References

[1] Balsano C., Alisi A., Brunetto M.R., Invernizzi P., Burra P., Piscaglia F., Special Interest Group Artificial Intelligence and Liver Diseases, Italian Association for the Study of the Liver (AISF) (2022). The application of artificial intelligence in hepatology: A systematic review. Dig. Liver Dis..

[2] Cabitza F., Campagner A., Balsano C. (2020). Bridging the “last mile” gap between AI implementation and operation: “data awareness” that matters. Ann. Transl. Med..

[3] Wong G.L.H., Yuen P.C., Ma A.J., Chan A.W.H., Leung H.H.W., Wong V.W.S. (2021). Artificial Intelligence in prediction of non-alcoholic fatty liver disease and fibrosis. J. Gastroenterol. Hepatol..

[4] Nam D., Chapiro J., Paradis V., Seraphin T.P., Kather J.N. (2022). Artificial Intelligence in liver diseases: Improving diagnostics, prognostics, and response prediction. JHEP Rep..

[5] European Association for the Study of the Liver (2021). EASL Clinical Practice Guidelines on non-invasive tests for evaluation of liver disease severity and prognosis—2021 update. J. Hepatol..

[6] Pickhardt P.J., Malecki K., Kloke J., Lubner M.G. (2016). Accuracy of Liver Surface Nodularity Quantification on MDCT as a Noninvasive Biomarker for Staging Hepatic Fibrosis. AJR Am. J. Roentgenol..

[7] Park H.J., Lee S.S., Park B., Yun J., Sung Y.S., Shim W.H., Shin Y.M., Kim S.Y., Lee S.J., Lee M.G. (2019). Radiomics Analysis of Gadoxetic Acid-enhanced MRI for Staging Liver Fibrosis. Radiology.

[8] Lili H., Li H., Dudley J., Maloney T.C., Brady S.L., Somasundaram E., Trout A., Dillman J.R. (2019). Machine Learning Prediction of Liver Stiffness Using Clinical and T2-Weighted MRI Radiomic Data. Am. J. Roentgenol..

[9] Schawkat K., Ciritsis A., von Ulmenstein S., Honcharova-Biletska H., Jüngst C., Weber A., Gubler C., Mertens J., Reiner C.S. (2020). Diagnostic accuracy of texture analysis and machine learning for quantification of liver fibrosis in MRI: Correlation with MR elastography and histopathology. Eur. Radiol..

[10] Lee J.H., Joo I., Kang T.W., Paik Y.H., Sinn D.H., Ha S.Y., Kim K., Choi C., Lee G., Yi J. (2020). Deep learning with ultrasonography: Automated classification of liver fibrosis using a deep convolutional neural network. Eur. Radiol..

[11] Wang K., Lu X., Zhou H., Gao Y., Zheng J., Tong M., Wu C., Liu C., Huang L., Jiang T. (2019). Deep learning Radiomics of shear wave elastography significantly improved diagnostic performance for assessing liver fibrosis in chronic hepatitis B: A prospective multicentre study. Gut.

[12] Gatos I., Tsantis S., Spiliopoulos S., Karnabatidis D., Theotokas I., Zoumpoulis P., Loupas T., Hazle J.D., Kagadis G.C. (2019). Temporal stability assessment in shear wave elasticity images validated by deep learning neural network for chronic liver disease fibrosis stage assessment. Med. Phys..

[13] Choi K.J., Jang J.K., Lee S.S., Sung Y.S., Shim W.H., Kim H.S., Yun J., Choi J.Y., Lee Y., Kang B.K. (2018). Development and Validation of a Deep Learning System for Staging Liver Fibrosis by Using Contrast Agent-enhanced CT Images in the Liver. Radiology.

[14] Yasaka K., Akai H., Kunimatsu A., Abe O., Kiryu S. (2018). Liver Fibrosis: Deep Convolutional Neural Network for Staging by Using Gadoxetic Acid-enhanced Hepatobiliary Phase MR Images. Radiology.

[15] Yu Q., Huang Y., Li X., Pavlides M., Liu D., Luo H., Ding H., An W., Liu F., Zuo C. (2022). An imaging-based artificial intelligence model for non-invasive grading of hepatic venous pressure gradient in cirrhotic portal hypertension. Cell Rep. Med..

[16] Wang J., Wang Z., Chen M., Xiao Y., Chen S., Wu L., Yao L., Jiang X., Li J., Xu M. (2022). An interpretable artificial intelligence system for detecting risk factors of gastroesophageal variceal bleeding. NPJ Digit. Med..

[17] Estes C., Razavi H., Loomba R., Younossi Z., Sanyal A.J. (2018). Modeling the epidemic of nonalcoholic fatty liver disease demonstrates an exponential increase in burden of disease. Hepatology.

[18] Fialoke S., Malarstig A., Miller M.R., Dumitriu A. (2018). Application of Machine Learning Methods to Predict Non-Alcoholic Steatohepatitis (NASH) in Non-Alcoholic Fatty Liver (NAFL) Patients. AMIA Annu. Symp. Proc..

[19] Liu F., Goh G.B., Tiniakos D., Wee A., Leow W.Q., Zhao J.M., Rao H.Y., Wang X.X., Wang Q., Wan W.K. (2020). qFIBS: An Automated Technique for Quantitative Evaluation of Fibrosis, Inflammation, Ballooning, and Steatosis in Patients with Nonalcoholic Steatohepatitis. Hepatology.

[20] Forlano R., Mullish B.H., Giannakeas N., Maurice J.B., Angkathunyakul N., Lloyd J., Tzallas A.T., Tsipouras M., Yee M., Thursz M.R. (2020). High-Throughput, Machine Learning–Based Quantification of Steatosis, Inflammation, Ballooning, and Fibrosis in Biopsies from Patients with Nonalcoholic Fatty Liver Disease. Clin. Gastroenterol. Hepatol..

[21] Taylor-Weiner A., Pokkalla H., Han L., Han L., Jia C., Huss R., Chung C., Elliott H., Glass B., Pethia K. (2021). A Machine Learning Approach Enables Quantitative Measurement of Liver Histology and Disease Monitoring in NASH. Hepatology.

[22] Greenspan H., Van Ginneken B., Summers R.M. (2016). Guest editorial deep learning in medical imaging: Overview and future promise of an exciting new technique. IEEE Trans. Med. Imaging.

[23] Hamm C.A., Wang C.J., Savic L.J., Ferrante M., Schobert I., Schlachter T., Lin M., Duncan J.S., Weinreb J.C., Chapiro J. (2019). Deep learning for liver tumor diagnosis part I: Development of a convolutional neural network classifier for multi-phasic MRI. Eur. Radiol..

[24] Olden J.D., Jackson D.A. (2002). Illuminating the “black box”: A randomization approach for understanding variable contributions in artificial neural networks. Ecol. Modell..

[25] Kiczales G. (1996). Beyond the black box: Open implementation. IEEE Softw..

[26] Ben-Cohen A., Klang E., Diamant I., Rozendorn N., Raskin S.P., Konen E., Amitai M.M., Greenspan H. (2017). CT Image-based Decision Support System for Categorization of Liver Metastases Into Primary Cancer Sites: Initial Results. Acad. Radiol..

[27] Wei S., Han Y., Zeng H., Ye S., Cheng J., Chai F., Wei J., Zhang J., Hong N., Bao Y. (2021). Radiomics diagnosed histopathological growth pattern in prediction of response and 1-year progression free survival for colorectal liver metastases patients treated with bevacizumab containing chemotherapy. Eur. J. Radiol..

[28] Dercle L., Lu L., Schwartz L.H., Qian M., Tejpar S., Eggleton P., Zhao B., Piessevaux H. (2020). Radiomics Response Signature for Identification of Metastatic Colorectal Cancer Sensitive to Therapies Targeting EGFR Pathway. J. Natl. Cancer Inst..

[29] Cheng S., Jin Z., Xue H. (2021). Assessment of Response to Chemotherapy in Pancreatic Cancer with Liver Metastasis: CT Texture as a Predictive Biomarker. Diagnostics.

[30] Shan Q.Y., Hu H.T., Feng S.T., Peng Z.P., Chen S.L., Zhou Q., Li X., Xie X.Y., Lu M.D., Wang W. (2019). CT-based peritumoral radiomics signatures to predict early recurrence in hepatocellular carcinoma after curative tumor resection or ablation. Cancer Imaging.

[31] Yang C., Huang M., Li S., Chen J., Yang Y., Qin N., Huang D., Shu J. (2020). Radiomics model of magnetic resonance imaging for predicting pathological grading and lymph node metastases of extra-hepatic cholangiocarcinoma. Cancer Lett..

[32] Liang W., Xu L., Yang P., Zhang L., Wan D., Huang Q., Niu T., Chen F. (2018). Novel nomogram for preoperative prediction of early recurrence in intrahepatic cholangiocarcinoma. Front. Oncol..

[33] Ji G.W., Zhu F.P., Zhang Y.D., Liu X.S., Wu F.Y., Wang K., Xia Y.X., Zhang Y.D., Jiang W.J., Li X.C. (2019). A radiomics approach to predict lymph node metastasis and clinical outcome of intrahepatic cholangiocarcinoma. Eur. Radiol..

[34] Xu L., Yang P., Liang W., Liu W., Wang W., Luo C., Wang J., Peng Z., Xing L., Huang M. (2019). A radiomics approach based on support vector machine using MR images for preoperative lymph node status evaluation in intrahepatic cholangiocarcinoma. Theranostics.

[35] Peng Y.T., Zhou C.Y., Lin P., Wen D.Y., Wang X.D., Zhong X.Z., Pan D.H., Que Q., Li X., Chen L. (2020). Preoperative ultrasound radiomics signatures for noninvasive evaluation of biological characteristics of intrahepatic cholangiocarcinoma. Acad. Radiol..

[36] Chu H., Liu Z., Liang W., Zhou Q., Zhang Y., Lei K., Tang M., Cao Y., Chen S., Peng S. (2021). Radiomics using CT images for preoperative prediction of futile resection in intrahepatic cholangiocarcinoma. Eur. Radiol..

[37] Vivanti R., Szeskin A., Lev-Cohain N., Sosna J., Joskowicz L. (2017). Automatic detection of new tumors and tumor burden evaluation in longitudinal liver CT scan studies. Int. J. Comput. Assist. Radiol. Surg..

[38] Tiyarattanachai T., Susantitaphong P., Marukatat S., Sukcharoen S., Yimsawad S., Chaichuen O., Komoltri C., Tiranathanagul K., Avihingsanon Y., Jutivorakool K. (2021). Development and validation of artificial intelligence to detect and diagnose focal liver lesions from ultrasonography still images. PLoS ONE.

[39] Kim J., Min J.H., Kim S.K., Shin S.-Y., Lee M.W. (2020). Detection of hepatocellular carcinoma in contrast-enhanced magnetic resonance imaging using a deep learning classifier: A multi-center retrospective study. Sci. Rep..

[40] Hassan T.M., Elmogy M., Sallam E.-S. (2017). Diagnosis of focal liver diseases based on deep learning technique for ultrasound images. Arab. J. Sci. Eng..

[41] Yasaka K., Akai H., Abe O., Kiryu S. (2018). Deep learning with convolutional neural network for differentiation of liver masses at dynamic contrast-enhanced CT: A preliminary study. Radiology.

